# Differential patterns of diversity at neutral and adaptive loci in endangered *Rhodeus pseudosericeus* populations

**DOI:** 10.1038/s41598-021-95385-w

**Published:** 2021-08-05

**Authors:** Hari Won, Hyung-Bae Jeon, Dong-Young Kim, Ho Young Suk

**Affiliations:** 1 Department of Life Sciences, Yeungnam University, Gyeongsan, Gyeongsangbuk-do South Korea; 2 Department of Biology, Concordia University, 7141 Sherbrooke W., Montreal, QC H4B 1R6 Canada

**Keywords:** Evolution, Genetics

## Abstract

Given the fact that threatened species are often composed of isolated small populations, spatial continuity or demography of the populations may be major factors that have shaped the species’ genetic diversity. Thus, neutral loci have been the most commonly-used markers in conservation genetics. However, the populations under the influence of different environmental factors may have evolved in response to different selective pressures, which cannot be fully reflected in neutral genetic variation. *Rhodeus pseudosericeus*, a bitterling species (Acheilognathidae; Cypriniformes) endemic to the Korean Peninsula, are only found in some limited areas of three rivers, Daecheon, Han and Muhan, that flow into the west coast. Here, we genotyped 24 microsatellite loci and two loci (DAB1 and DAB3) of MHC class II peptide-binding β1 domain for 222 individuals collected from seven populations. Our microsatellite analysis revealed distinctive differentiation between the populations of Daecheon and Muhan Rivers and the Han River populations, and populations were structured into two subgroups within the Han River. Apparent positive selection signatures were found in the peptide-binding residues (PBRs) of the MHC loci. The allelic distribution of MHC showed a degree of differentiation between the populations of Daecheon and Muhan Rivers and the Han River populations, partially similar to the results obtained for microsatellites, however showed rather complex patterns among populations in the Han River. Considering the apparent differences in the distribution of supertypes obtained based on the physicochemical differences induced by the polymorphisms of these PBRs, the differentiation in DAB1 between the two regional groups may result in the differences in immune function. No differentiation between these two regions was observed in the supertyping of DAB3, probably indicating that only DAB1 was associated with the response to locally specialized antigenic peptides.

## Introduction

Freshwater fish species, which make up nearly a quarter to half of the world's vertebrate diversity^1,2^, are recently disappearing more rapidly than terrestrial or marine animal species^3^. The main reason for the contemporary loss of freshwater fish diversity is that freshwater ecosystems are particularly sensitive to the effects of anthropogenic activities, including habitat loss, alien species introduction and pollution, primarily due to their insular and fragmented nature^3–5^. No matter how global wildlife conservation efforts are underway, it seems impossible to stop the widespread decline in freshwater fish diversity^2,6^. Considering this situation, it is effective to focus more on the conservation of species specifically vulnerable to extinction, for example species with small native ranges.

One of the most important steps to consider before establishing management strategies for a species is to understand its genetic diversity and population structure^7–9^. Given that threatened species are often composed of small fragmented populations^10^, spatial continuity or demography of the populations may be major factors that have shaped the species’ current genetic diversity and characteristics^11,12^. Thus, neutral loci with signatures of such spatio-temporal histories are the most commonly used markers in conservation genetics^13^. However, the populations under the influence of different environmental factors may have evolved in response to different selective pressures^14–18^, which cannot be fully reflected in neutral genetic variation^19^.

The peptide-binding region in the major histocompatibility complex (MHC) molecules has been considered as one of the widely used genetic marker in the context of local adaptation^20,21^. MHC molecules are divided into class I and class II^22,23^. All types of nucleated cells express MHC class I, whereas MHC class II molecules are only found on specialized antigen-presenting cells^22,23^. MHC class I presents a peptide fragment of cytoplasmic proteins or pathogens to the receptor of cytotoxic T cells that express CD8^22,23^. A peptide fragment loaded by MHC class II is derived from the lysosomal process of proteins or pathogens internalized by endocytosis and presented to the receptor of helper T cells that express CD4^22,23^. An enormous polymorphism is found in the domain to which a peptide bind^24^. The diversity of this domain confers a variety of peptide-binding repertoire, and is known to be the historical consequences of co-evolutionary arms race with the local pathogens^24–27^. Local populations living under distinct composition of pathogens are thus expected to have a different MHC allelic composition^25,27^.

**Figure 1:**
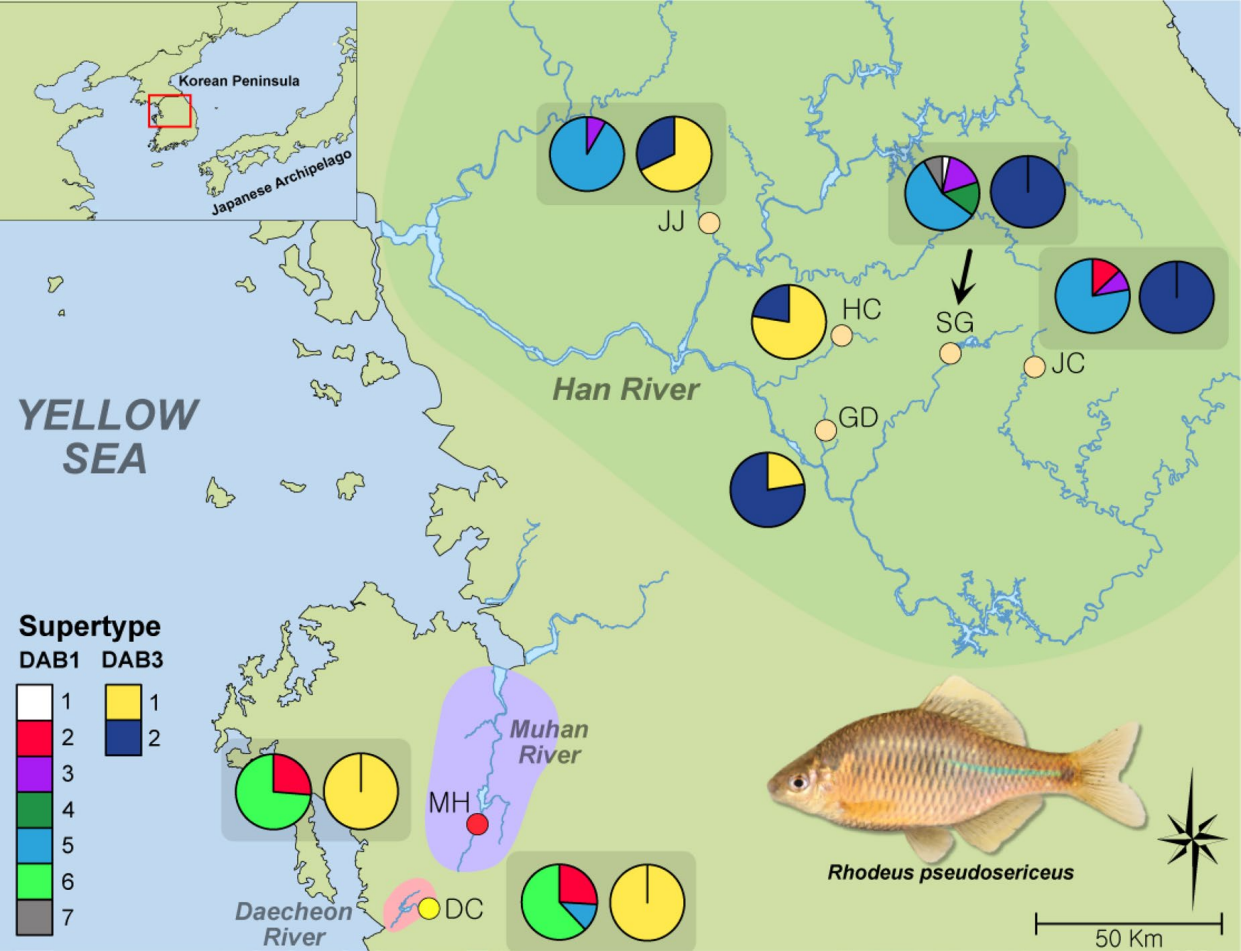
The collection site information (population) of *Rhodeus pseudosericeus* with a map in the square left above showing the locations of the Korean Peninsula and the surrounding areas. The locations of the seven *R. pseudosericeus* populations collected from the Daecheon River (DC), Muhan River (MH) and Han River (GD, HC, JC, JJ and SG) were indicated with the pie graphs showing the frequencies of DAB1 and DAB3 supertypes. on the GPS coordinates of the populations are as follows: DC (36°23′28.00″N, 126°39′48.00″E), MH (36°27′38.63″N, 126°44′15.91″E), GD (37°20′2.00″N, 127°40′37.00″E), HC (37°30′40.71″N, 127°38′47.83″E), JC (37°25′52.32″N, 128°10′47.16″E), JJ (37°44′50.65″N, 127°26′4.05″E) and SG (37°32′29.17″N, 127°58′9.43″E). The shape of the map was downloaded from http://www.biz-gis.com. The map was processed using QGIS 3.4 (Open Source for Geospatial Foundation Project; http://qgis.osgeo.org) and converted into a vector image that was visualized with Affinity Designer 1.8.5. All URLs, materials and applications used to create this map are open to non-commercial use without permission.

*Rhodeus pseudosericeus*, a bitterling species (Acheilognathidae; Cypriniformes) endemic to the western Korean Peninsula, is legally designated as an endangered species (class II) under the Protection Act of Wild Fauna and Flora by the Ministry of Environment, South Korea^28^. Like other bitterling species, *R. pseudosericeus* spawns only into living unionid mussels^28^. Populations of this species are only found in some limited areas of three rivers, Daecheon, Han and Muhan that flow into the west coast^28^ (Fig. 1). Although present in a very small distributional range, the populations of this species are exposed to two completely distinct environments. The Daecheon and Muhan Rivers are small streams with a length of about 20 and 50 km, respectively, while the Han River is a large river about 500 km long, beginning at around the eastern end of the peninsula^29^. The Han River’s wide water system and mountainous terrain provides ecological features never found in the Daecheon and Muhan Rivers, and distinct selection pressures (i.e., pathogen fauna) would have worked between Han and Daecheon-Muhan. Only a single genetic analysis for this species, using the mitochondrial cytochrome *b* gene, has been performed to date^28^. In that study, the populations in the Han River showed a slightly distinct haplotype composition from the Daecheon and Muhan Rivers that were genetically similar to each other^28^.

Here, we genotyped 24 microsatellite loci and two loci (DAB1 and DAB3) of MHC class II β1 domain (peptide-binding domain) for 222 *R. pseudosericeus* individuals collected from seven populations (Fig. 1). First, our microsatellite data, along with existing mitochondrial cytochrome *b* data^28^, are expected to provide insight into the neutral history that have shaped these populations. Second, we attempted to determine the extent to which the historical processes reflected in the neutral genetic variation have also contributed to MHC genetic variation. The MHC alleles detected were grouped into immunological supertypes based on the similarity in the physicochemical properties of the peptide-binding residues. Finally, based on the distribution pattern of MHC supertypes, we attempted to find evidence for parasite-mediated selection acting on *R. pseudosericeus* populations. Our research will provide the essential data to establish the management units of severely endangered *R. pseudosericeus* and future reconstruction plans of the native populations.

## Results

### Microsatellite analysis

The level of microsatellite diversity varied greatly across loci, with the number of alleles per locus ranging from 3 (*TS27*) to 24 (*TS76*), and the expected heterozygosity ranging from 0.233 (*TS27*) to 0.751 (*TS76*; Supplementary Table S1). At the locus level, five loci (*TS06*, *TS18*, *TS19, TS22* and *TS81*) deviated significantly from the expectation of HWE (Supplementary Table S1). The data of these loci were excluded. No significant departure from HWE was detected at the population level (Table 1). When comparing genetic diversity by population, the three populations of the Han River, JJ, HC and JC, showed rather lower values than others (Table 1). No evidence of contemporary population bottleneck was found from the mode-shift in allele class distribution nor Wilcoxon signed-rank test (following Bonferroni adjustment; data not shown). Based on the *M*-ratio estimation, the two Han River populations, GD and JC, appear to have experienced a marked historical demographic decline (Table 1).

**Table 1:** Summary of seven *Rhodeus pseudosericeus* populations analyzed in this study and the microsatellite and MHC (DAB1 and DAB3) diversity estimates. The data include population (ID), total number of individuals analyzed (*N*), total number of alleles per locus (*A*), allelic richness (*A*_R_), observed (*H*_O_) and expected (*H*_E_) heterozygosities and *M*-ratio (*M*) for microsatellites and number of alleles, allelic (haplotypic; *A*_M_) diversity (*A*_d_) and nucleotide diversity (*π*) for DAB1 and DAB3.

Population	Microsatellites						DAB1		DAB3	
	*N*	*A*	*A*_R_	*M*	*H*_O_	*H*_E_	*A*_M_	*A*_d_ (π)	*A*_M_	*A*_d_ (π)
Daecheon (DC)	32	5.053	4.970	0.78	0.579	0.600	4	0.716 (0.048)	2	0.171 (0.014)
Muhan (MH)	29	4.895	4.894	0.68	0.612	0.612	5	0.613 (0.023)	1	0 (0)
**Han River**										
Jojong (JJ)	30	2.842	2.833	0.81	0.326	0.311	3	0.163 (0.010)	2	0.444 (0.027)
Heuk (HC)	29	3.368	3.368	0.70	0.425	0.447	–	–	2	0.373 (0.023)
Geumdang (GD)	33	4.579	4.493	0.55	0.541	0.574	–	–	3	0.427 (0.203)
Seom (SG)	32	5.578	5.541	0.68	0.513	0.551	8	0.695 (0.056)	2	0.329 (0.006)
Jucheon (JC)	37	2.263	2.215	0.53	0.378	0.378	6	0.712 (0.018)	1	0 (0)

**Figure 2:**
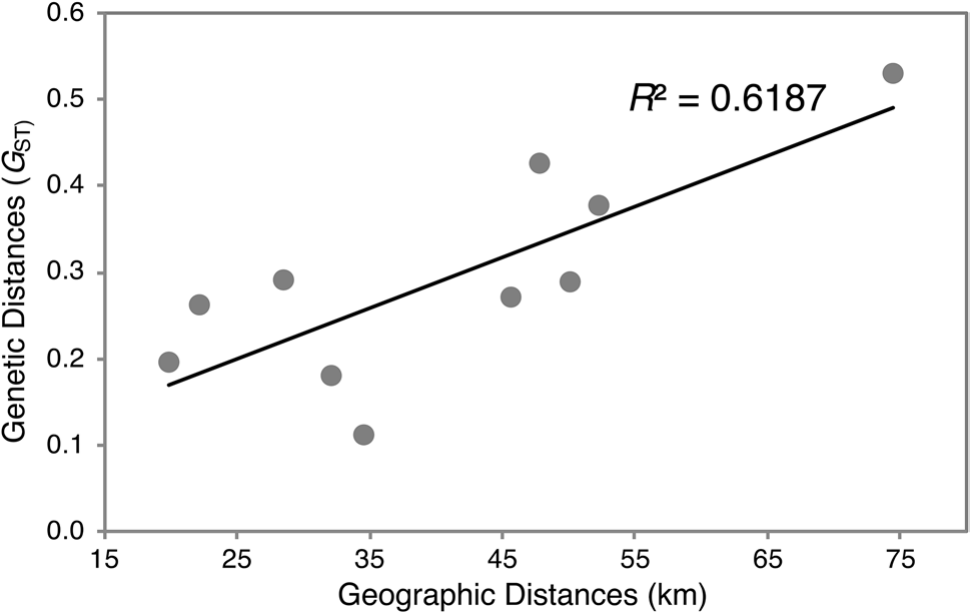
A plot showing that the genetic distance (*G*_ST_) is determined by the spatial distance (along the water way) between the five Han River populations of *Rhodeus pseudosericeus*.

**Figure 3:**
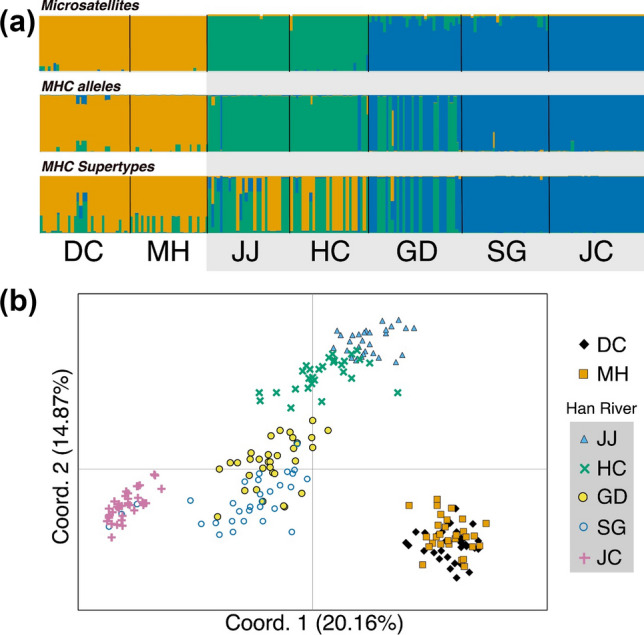
(**a**) Population genetic structure of *Rhodeus pseudosericeus* estimated from microsatellite genotyping and MHC allelic and supertypic data. The population structure estimated in Structure using the most reliable number of clusters predicted by delta *K* method (*K* = 3 for all three; see Supplementary Fig. S1). (**b**) Bi-dimensional plot of the principal component analysis (PCA) showing genetic differentiation based on microsatellite pairwise-*F*_ST_ values among seven populations of *R. pseudosericeus* from the Korean Peninsula. See Table 1 or Fig. 1 for locality codes. The population IDs of the Han River were shaded in the pictures.

The overall level of genetic differentiation among populations was substantial with global *F*_ST_ = 0.324. and *R*_ST_ = 0.338. The Daecheon and Muhan River populations (DC and MH) were genetically close with each other, whereas these two river populations were genetically distinct from the Han River populations (Table 2). Despite being populations located in the same water system, the degree of genetic differentiation among the Han River populations was rather high with average pairwise-*F*_ST_ and -*R*_ST_ of 0.286 (0.112–0.533) and 0.234 (0.102–0.431), respectively (Table 2). The genetic differentiations among the Han River populations were positively correlated with the geographic distances (Fig. 2). Our Bayesian clustering analysis also visualized the pattern of genetic structuring among *R. pseudosericeus* populations shown in the pairwise-*F*_ST_ and -*R*_ST_ analysis. Distinctive differentiation was observed between DC-MH and the Han River populations, and the Han River populations were likely subdivided into two groups (JJ-HC, JC-SG-GD; Fig. 3a). Upon PCA analysis, these three genetic groups were also identified (Fig. 3b).

**Table 2:** Pairwise microsatellite genetic differentiation of seven *Rhodeus pseudosericeus* populations from the Korean Peninsula. Estimates of *F*_ST_ appear above the diagonal and estimates of *R*_ST_ appear below the diagonal. All comparisons were significantly different from zero (*P* < 0.05).

	DC	MH	Han River populations				
			JJ	HC	GD	SG	JC
DC		0.114	0.390	0.338	0.259	0.290	0.474
MH	0.114		0.370	0.336	0.269	0.307	0.470
JJ	0.426	0.363		0.179	0.285	0.375	0.533
HC	0.432	0.316	0.205		0.112	0.289	0.430
GD	0.464	0.308	0.251	0.102		0.118	0.276
SG	0.399	0.286	0.146	0.106	0.109		0.266
JC	0.515	0.463	0.393	0.284	0.431	0.322

### MHC analysis

Twenty-one DAB1 and five DAB3 alleles were determined through our validation processes. These alleles were also confirmed to be the correct β1 domain region based on the comparison with mammalian classical DRB sequences. The overall level of non-synonymous substitution (*dN*/*dS*) was significantly high only in DAB3 (Supplementary Table S2). However, significance was found in both DAB1 and DAB3, when only considering peptide binding residues (PBRs; Supplementary Table S2). Two positive selection models, M2a and M8, seemed to be significantly better suited for our DAB1 and DAB3 sequence data than nearly neutral (M1a) and β distribution (M7), respectively (Supplementary Table S3, Supplementary Table S4 and Supplementary Table S5). From the Bayes empirical Bayes (BEB) analysis in CODEML, the significant signature of positive selection was found from fourteen codons in DAB1 (Supplementary Table S3 and Table 3) and nine codons in DAB3 (Supplementary Table S4 and Table 4). Of them, ten and seven were PBRs of DAB1 (Table 3) and DAB3 (Table 4), respectively. Based on the tests implemented in DataMonkey web server (FEL, FURBAR and MEME), the signature of positive selection was found in a much smaller number of codons (Tables 3 and 4).

**Table 3:** Identification of the codons in DAB1 showing the signature of positive selection predicted based on the five different models implemented RDP (using HyPhy package) and CODEML. The codons predicted to be PBR were highlighted by bold.

	**9**	10	11	12	13	14	15	16	17	18	19	20	21	22	23	24	**25**	26	**27**	28	29	30	31	32	33	**34**	**35**	36	37	38	39	40	41	42	43	**44**	45
FEL	+																																				
MEME	+																		+																		
FUBAR	+																		+																	+	
M2a	+														+				+							+										+	
M8	+														+				+							+										+	

	46	47	48	49	50	51	52	**53**	54	55	56	**57**	**58**	59	60	61	**62**	63	64	**65**	66	**67**	**68**	69	70	**71**	72	73	74	**75**	76	77	**78**	**79**	80	81	
FEL																						+				+				+							
MEME												+	+					+	+			+				+											
FUBAR																			+			+				+			+	+							
M2a					+			+				+	+						+							+	+		+	+							
M8					+			+				+	+						+							+	+		+	+

**Table 4:** Identification of the codons in DAB3 showing the signature of positive selection predicted based on the five different models implemented RDP (using HyPhy package) and CODEML. The codons predicted to be PBR were highlighted by bold.

	**9**	10	11	12	13	14	15	16	17	18	19	20	21	22	23	24	**25**	26	**27**	28	29	30	31	32	33	**34**	**35**	36	37	38	39	40	41	42	43	**44**	45	46
FEL																																						
MEME																																						
FUBAR																																						
M2a	+																		+																	+		
M8	+																		+																	+		

	47	48	49	50	51	52	**53**	54	55	56	**57**	**58**	59	60	61	**62**	63	64	**65**	66	**67**	**68**	69	70	**71**	72	73	74	**75**	76	77	**78**	**79**	80	81	82	83	
FEL																																						
MEME																																						
FUBAR																					+																	
M2a							+					+						+				+															+	
M8							+					+						+				+						+									+	

In the analysis of DAB1, GD and HC were excluded from inter-populational comparisons, because the PCR amplification was not successful in most individuals from these populations. Thus, two alleles that appeared only in these populations were also excluded from the comparisons. Populations DC and MH were similar in the composition of DAB1 alleles (Supplementary Table S6 and Table S7). However, these two populations rarely share alleles with the Han River populations (Supplementary Table S6 and Table S7). Alleles of DAB1 were allocated into seven supertypes (Fig. 1; Supplementary Table S8). The difference in the distribution of supertypes was evidently revealed between DC-MH and the Han River populations (Fig. 1; Supplementary Table S8). In the analysis of DAB3, there was no shared alleles between DC-MH and the Han River populations (Supplementary Table S6). Despite being located in the same water system, the five Han River populations could be grouped into two clusters, HC + JJ and GD + JC + SD (Supplementary Table S6and Table S7). The distribution of the DAB3 supertypes was slightly structured between DC-MH and the Han River populations (Fig. 1; Supplementary Table S8). The relatively downstream populations in the Han River, GD, HC and JJ, shared a supertype of DC-MH, whereas populations JC and SG showed a single DAB3 supertype completely distinct from DC-MH (Fig. 1; Supplementary Table S8). The difference between DC-MH and Han River populations was clearly revealed in Bayesian Structure analysis, which was calculated by comprehensively considering the distribution of alleles of DAB1 and -3 (Fig. 3a). A clear genetic structure was found among Han River populations, though population GD contained the genetic features of both HC-JJ and JC-SD, slightly different from the microsatellite results (Fig. 3a). As a result of the Structure synthesizing the supertypes of DAB1 and -3, HC-JJ and DC-MH were shared to some extent, population GD also contained the features of both HC-JJ and JC-SD (Fig. 3a).

## Discussion

This study was performed to identify the signatures of the historical processes that have shaped the contemporary distribution and genetic pattern of *Rhodeus pseudosericeus*. In the previous report of mitochondrial cytochrome *b* sequence analysis for this species, the populations DC and MH were clearly distinguished from those of the Han River^28^. In addition, the populations of the Han River showed relatively low levels of genetic diversity^28^. Findings based on our data assist in disentangling historical events of the populations at different timescales, given that mitochondrial and microsatellite markers have different rates of evolution^30–33^. According to a previous study in *R. notatus*, another bitterling species distributed throughout the Korean Peninsula, rivers on the west coast may have formed confluence at least intermittently when sea level was lower than at present^34^. Considering both the previous mitochondrial data and our data, the genetic similarity of the two geographically close populations, DC and MH, suggests the possibility that the rivers of these two populations shared an estuary region in the past. However, the genetic separation between DC + MH and the Han River in the mitochondrial results^28^ provides insight into the long history of isolation between ancient populations of *R. pseudosericeus* during the colonization processes. Our microsatellite genotyping results also provided information about relatively recent demographic changes after colonization of the populations.

Given that *R. pseudosericeus* is an endangered species, the diversity of this species at the population level is important. The microsatellite diversity of *R. pseudosericeus* was revealed to be nearly the same as the average of freshwater fish species reported previously^35,36^. The microsatellite diversity of some bitterling species inhabiting the Korean Peninsula has also been studied. However, studies of bitterling species so far do not show a correlation between the abundance and genetic diversity of natural populations. For example, the average microsatellite diversity of *R. notatus*, which has a broad distribution and is not a legally protected species, was even lower than that of *R. pseudosericeus*^34^. In contrast, *Tanakia somjinensis*, one of the endangered bitterlings in South Korea, showed a tremendous level of microsatellite diversity, though this species is observed only in a single river over the world^37^.

The average microsatellite diversity of the Han River populations was lower than that of populations DC or MH. A similar pattern was found in the investigation based on mitochondrial cytochrome *b* sequences^28^. In particular, populations GD and JC showed a signature of distinct historical demographic decline, when judged based on our *M*-ratio data. No evidence of contemporary bottleneck was detected. Based on the current distribution of *R. pseudosericeus*, two inferences can be made for the low genetic diversity of the Han River populations. First, the paleo-climatic and geographic history of the freshwater system on the Korean Peninsula should be considered. When the sea level before the end of the glacial period was about 120 m lower than at present, the west coast of the peninsula was a part of the paleo-Yellow River flowing through the mainland of China, and the Han, Daecheon and Muhan River formed confluences at least intermittently with the paleo-Yellow River^28^. In the Daecheon and Muhan River, early populations would have been able to quickly and easily occupy and thrive in appropriate habitats, because these two rivers were relatively short with habitats suitable for this species, i.e*.*, tributary environments. On the contrary, the populations in very long Han River may have been formed relatively recently by a small number of individuals originated from the estuary. The repetitive genetic drift experienced during such a process may have shaped the genetic characteristics of the current Han River populations. Second, the environment provided from the Han River may not be suitable for the survival of *Rhodeus* bitterling, compared to that of the southern rivers on the peninsula. Although no ecological data have yet been performed to support this hypothesis, it is worth noting the genetic investigation of *R. notatus*, where the genetic diversity of the Han River population was significantly lower than those of other river populations^34^.

In the very long Han River, population genetic structure was also clearly revealed in our study. The reason for this genetic differentiation among populations within a single water body, where there are no natural landscape barriers to population migration, may be attributed to the effects of sequential colonization and genetic drift, as mentioned above. There is one more factor to consider. Our results showed a clear positive correlation between geographical and genetic distances between populations within the Han River, which suggests that it is unlikely for *R. pseudosericeus* inhabiting tributary regions to perform long distance migration along the main stem of the river.

The DAB1 and DAB3 sequences used in this study can be regarded as the functional and classical MHC class II β1 alleles, because major amino acids that are considered necessary for adaptive immune function were identified when comparing with other teleost or vertebrate species^21,38–42^. In both DAB1 and DAB3, the signature of positive selection was clearly detected. Although classical MHC loci are generally known to have high allelic polymorphism, low levels of diversity were detected in both DAB loci of *R. pseudosericeus*. The number of alleles per population ranged from three to eight for DAB1 and from one to three for DAB3. To date, the diversity analysis of DAB sequences has been performed for three bitterling species. One of those species, *R. ocellatus* should not be compared with our findings, because the study^43^ was only designed for behavioral testing of this species with no comprehensive survey of the populations for the diversity estimation of DAB loci. Second, only 16 DAB alleles have been detected in *Pseudorhodeus tanago*, an endangered endemic bitterling species to Japan^44^. Finally, a recent study of *R. sinensis* revealed a huge level of MHC diversity, with 140 alleles detected from DAB1 and DAB3^21^. However, it should be considered that the populations of *R. sinensis* are very common in most major river basins on the Korean Peninsula^21^. Although it is difficult to make any conclusive statements, the low MHC diversity of *R. pseudosericeus* seems to be associated with the narrow distribution range and the isolation of populations. In *R. pseudosericeus*, the diversity of DAB3 was found to be extremely low compared to DAB1, which may be a characteristic of cyprinids or *Rhodeus*^21^, though not investigated in various species.

Parasite-mediated balancing selection, appearing as frequency-dependent selection, is the most well-known evolutionary force that drives the diversity of MHC genes^25,26,45–47^. Given the low intra-population DAB allelic diversity, it can also be predicted that there may be a strong effect of genetic drift, probably because of the small effective population sizes. Since almost perfectly distinct allelic divergence was found in both DAB1 and DAB3 between populations DC-MH and the Han River populations, the positive selection signature found here might be the result of directional selection that has worked differently on these two regional groups. However, it can not be argued that only the directional selection effect worked on the allelic divergence of MHC between these two regional groups. Given that these two regions have been almost completely isolated, as supported by mitochondrial and microsatellite markers, this genetic differentiation may simply be due to a history of independent mutation accumulation and genetic drift. Considering the genetic structure of MHC among populations in the Han River, it is somewhat insufficient to understand that neutral forces, such as gene flow or genetic drift, were the only determinants. For example, the allelic composition of population GD was found to have both extreme compositions of Han River populations simultaneously, indicating that the influence of the balancing selection force has been at work to some extent^47^, given that this population is not spatially in a location where two different genetic makeups would meet. Since our study was not designed to estimate the relative contribution of balancing and directional selection pressures to the pattern of genetic variance, yet, it is premature to draw any conclusions based on the data obtained here.

In order to determine whether this differentiation of MHC is related to differences in immunological function, alleles of DAB1 and DAB3 were translated into functional supertypes, respectively. In DAB1, supertypes were shared only at low frequency between these two regional groups, suggesting that phenotypic differences in DAB1 between the two groups may be linked to the ability to bind specific pathogen-derived peptides and consequently pathogen resistance. In the case of DAB3, in contrast, some of the Han River populations shared the same supertype (supertype 1; Fig. 1 and MHC supertypes in Fig. 3) with populations DC and MH. Variation of DAB1 may thus be responsible for the response to locally specialized antigenic peptides, whereas DAB3 may be associated with the detection of a wide range of pathogens occurring without regional differences. Although no ecological analysis was performed in our study, the locations of the populations with high frequency of DAB3 supertype 1 were the water areas flowing along the plains, while those with high frequency of supertype 2 were surrounded by mountainous terrain, based solely on our field sampling experience. In the data comprehensively considering the supertypes of DAB1 and 3, a distinct structure was found between HC-JJ and JC-SG, and the population GD was found to have both of these two different supertype compositions. In this respect, different selection responses exist for pathogen fauna even within the Han River, and it can be considered that there is a balancing selection pressure within the population. Therefore, it is necessary to analyze the pathogen fauna in the areas where the populations used in this study are located in future studies.

Overall, the MHC and microsatellite loci we investigated in *R. pseudosericeus* showed similar patterns of population genetic structure. Although the genetic structure among the populations of the Han River did not appear in DAB1 (probably due to the failure of amplification in two populations), the populations of the Han River in DAB3 were structured similarly to those revealed in the microsatellite loci. More importantly, the allelic structure in DAB3 was rather different from the pattern revealed by MHC supertyping. This may reflect a history of isolation among population groups within the Han River. As such, being able to measure the relative importance of neutral change and adaptation to the environment can be an important benefit of investigating adaptive loci. Considering these points, caution is required during the conservation management of the populations in the Han River.

Taken together, two main parts should be considered in the perspective of conservation and management. First, it should be noted that the genetic diversity of the Han River populations is relatively lower than that of the populations DC or MH. In addition, genetic features that appear to have been formed under the influence of genetic drift or demographic fluctuation were also revealed. A more detailed ecological survey of these species in the future will be beneficial for establishing conservation strategies. Second, our findings support that the populations of the Han River and the populations of the Daecheon and Muhan Rivers likely have specialized immune adaptations to different types of pathogens, based on the results of MHC supertyping. Therefore, these two regional groups need to be established as separate conservation units.

## Materials and methods

### Samples and DNA extraction

All *Rhodeus pseudosericeus* samples used for the genetic analysis were the fin clips removed from the specimens stored in the Department of Life Sciences at Yeungnam University, which were also used in a previous study^28^. These specimens were collected in accordance with the Inland Water Fisheries Act and Wildlife Protection and Management Act in the South Korea from seven populations of the Daecheon River (DC), Muhan River (MH) and Han River (GD, HC JC, JJ and SG; Table 1)^28^. Since stored specimens were used, no separate authorization procedure was required according to Yeungnam University regulations, but all experimental procedures were conducted in accordance with the guidelines by Yeungnam University Institutional Animal Care and Use Committee. Genomic DNA was isolated using a Wizard Genomic DNA purification kit (Promega, Madison, WI, USA).

### Microsatellite genotyping

To determine the population diversity and genetic structure of *R. pseudosericeus*, we tested the microsatellite loci known from other species in Acheilognathidae and then selected those with high PCR success rate and variability. We tested 13 and 110 microsatellites developed from *R. sinensis* (series RU)^48^ and *Tanakia somjinensis* (series TS)^37^, respectively. Through the tests using eight randomly selected *R. pseudosericeus* individuals, a total of 24 loci that showed good polymerase chain reaction (PCR) amplification efficiency and polymorphism visually observed in electrophoresis were selected to be used for genotyping in this study (Supplementary Table S1).

One of four different types of fluorescence, FAM, PET, VIC or NED was labelled at the 5′ end of each forward primer (Applied Biosystems, Waltham, MS, USA). PCR was performed using a 10 μl mixture comprising 1 μl of DNA extraction, 1 × *Taq* buffer (containing 2.5 mM MgCl_2_), 0.25 mM dNTPs, 1 μM of each primer and 0.25 units of *Taq* DNA polymerase (Solgent, Daejeon, South Korea). Thermal cycling using GenePro (Bioer, Hangzhou, China) was performed under the profile consisting of an initial denaturation at 95 °C for 5 min, 35 cycles of a denaturation at 95 °C for 30 s, an annealing at 58 °C for 30 s, an extension at 72 °C for 45 s, and a final extension at 72 °C for 10 min. The amplified products were genotyped on an ABI 3730xl Genetic Analyzer by Macrogen Inc (Seoul, South Korea).

### Microsatellite analysis

Genetic variability per locus and population was estimated with the number of alleles, allelic richness, observed heterozygosity, expected heterozygosity and fixation index (*F*_IS_) using Arlequin 3.5^49^, Fstat 2.9.3.2^50^ and Genepop 4.2^51^. The deviation of observed genotype frequencies from the expectation of Hardy–Weinberg equilibrium (HWE) was tested for each locus and each population using the Fisher’s exact test based on Markov chain parameters with 1000 batches and 10,000 iterations per batch^52^ in Genepop. Fisher’s exact test was also used in testing the existence of linkage disequilibrium between pairs of loci based on the Markov chain algorithm under the null hypothesis of independence using Genepop. Micro-Checker 2.2.3^53^ was used to check the likelihood of genotyping errors caused by the presence of null alleles or allele dropout.

For each population, two different methods were used to determine whether there was a signature of recent reduction in population size using Bottleneck 1.2.1^54^. First, it was examined whether the expected heterozygosity was significantly exceeded when the mutation-drift equilibrium was assumed compared to the HWE based on the Wilcoxon sign-rank test under the TPM (two phase model) with a setting of 90% stepwise mutations model (SMM) and 10% infinite alleles model (IAM). Second, mode-shift was examined to check if the distribution of allelic class deviated from the typical L shape^55,56^. The probability of a historical population size reduction was examined by whether the *M*-ratio, which is the mean ratio of the number of alleles to the range in allele size, was significantly lower than the commonly used threshold, 0.68^57^.

Three different methods were used to identify the genetic structure among *R. pseudosericeu*s populations. First, global *F*_ST_ and *R*_ST_ as well as pairwise-*F*_ST_ and pairwise-*R*_ST_ were estimated using Arlequin and Genepop. GenAlEx 6.5^58^ was applied to evaluate the relationship between pairwise genetic divergence and the geographic distance along the water way. The interpopulation geographic distance was estimated using Googlemap’s ‘Measure Distance’ function. Second, the pattern of population structuring was examined based on Bayesian framework using the software Structure 2.3.4^59^. The reliable numbers of genetically distinguishable clusters (*K*) were predicted by Δ*K*^60^ implemented in Structure Harvester v0.6.94^61^. Bayesian Structure analysis was performed for each *K*, with ten independent MCMC runs, each consisting of 4 × 10^5^ generations after a burn-in of 10^5^. Finally, principal component analysis (PCA) was performed using GenAlEx to visualize the relationship among populations at the individual level. Critical significance values of all statistics used were adjusted for multiple comparisons based on the Bonferroni procedure.

### MHC genotyping

We employed a two-step tailed PCR approach to construct the paired-end libraries for high throughput sequencing of MHC using MiSeq platform. As with most cypriniform species, MHC class IIB of bitterlings can be assigned into two evolutionarily distinct loci, DAB1 and DAB3^21^. DAB1 (212 bp) and DAB3 (227 bp) were amplified using the primer pairs RPMHCD1E2 (DAB1; forward: CAT GGA TAC TAC TRK TCT CGG TGG; reverse: AGC TGC CTG WYW KAK TTC AGC A) and RPMHCD3E2 (DAB3; forward: GAT GGA TAT TAT SAA TAC GAC AT; reverse: TGC TTT ATC ACG GAC AGC TGR GT), respectively, designed based on the relatively conserved sequences at both ends of the peptide-binding domain (β1 domain). Draft sequences of DAB1 and DAB3 obtained from four *R. pseudosericeu*s individuals of population DC, which was known to show the highest genetic diversity among the populations^28^, were used for the comparison with other species^21^ and the design of this primer set. Primers designed in a closely related species, *R. sinensis*^21^ were used to analyze this full DAB sequences. The forward and reverse primers of both RPMHCD1E2 and RPMHCD3E2 carried 5′ end overhang extension to provide a primer binding site for the second PCR and the pair-end sequencing on MiSeq platform (5′-ACA CTC TTT CCC TAC ACG ACG CTC TTC CGA TCT—forward primer; 5′-GTG ACT GGA GTT CAG ACG TGT GCT CTT CCG ATC T—reverse primer). The first PCR amplification was performed in a 25 μl reaction volume containing 2.5 μl template, 12.5 μl 2 × NEBNext Ultra II Q5 Mater Mix (New England Biolabs, Ipswich, MS, USA), 1.25 μl of each primer (10 μM) and 7.5 μl distilled H_2_O. The thermal cycling profile was as followed: an initial denaturation at 98 °C for 30 s, 30 cycles of a denaturation at 98 °C for 10 s, an annealing at 60 °C for 30 s and an extension at 72 °C for 30 s, and a final extension at 72 °C for 5 min. The first amplicons were sent to GenoTech Inc (Daejeon, South Korea) for the next steps including the second PCR for library preparation, MiSeq sequencing and data processing.

In the second PCR, the first amplicons were amplified using the forward and reverse primers tagged with a unique combination of 8 bp index sequence allowing the identification of each individual and an adaptor sequence allowing the final amplicons to hybridize to the MiSeq flowcells (Supplementary Table S9). The PCRs were performed in 50 μl volumes containing 5 μl template, 25 μl 2 × NEBNext Ultra II Q5 Mater Mix, 5 μl of each primer (10 μM) and 10 μl distilled H_2_O using the following settings: an initial denaturation at 98 °C for 30 s, 10 cycles of a denaturation at 98 °C for 10 s, an annealing at 55 °C for 30 s and an extension at 65 °C for 45 s, and a final extension at 65 °C for 5 min. The final PCR products were pooled in equal volumes, purified using AMPure XP beads (Beckman Coulter, Pasadena, CA, USA), and subjected to TapeStation HSD5000 (Agilent, Santa Clara, CA, USA) to check the purity, concentration and fragment size. The final library preparation was subjected to the pair-end sequencing mode of 300 bp read length in the Illumina MiSeq platform (Illumina, Inc., San Diego, CA, USA).

### MHC allele validation

We validated MHC alleles according to the protocol proposed in a previous study^62^. Resulting reads (DAB1: 5,419,573; DAB3: 8,022,886) were quality-filtered with CLC genomics workbench v6.5 (CLC Bio, Aarhus, Denmark) to remove the sequences having a Phred score less than Q30 or ambiguous nucleotides and the sequences more than five bp longer or shorter in length than expected. The 3,343,997 (DAB1) and 5,419,573 (DAB3) reads that passed these criteria were mapped to the draft DAB sequences obtained from four individuals in the population DC. Sequences that were completely mapped (DAB1: 99.97%; DAB3: 99.99%) were demultiplexed, and their adaptors and primer sequences were trimmed. During this process, sequences with incorrect adapters or primer sequences were manually removed. The sequences were subjected to two additional steps of validation. First, an amplicon must appear in more than one individual in the population to be recognized as an allele. The only exception was permitted for the amplicons that appeared only in one heterozygote individual. Second, a single amplicon should be supported with more than 1000 reads or more than the frequency of 0.1. This validation protocol can prevent chimeric sequences or PCR artifacts from being included in our analysis, although the possibility exists to slightly underestimate the diversity of MHC.

Since amplification was not successful for the DAB1 of many individuals from populations GD and HC, the DAB1 data of these two populations were excluded from the analysis. This may be due to the presence of mutations in the priming sites, and it is also possible that the presence of adaptor sequences for two-step PCR significantly lowered the efficiency of amplification in these populations. However, considering that the possibility of amplification of different loci cannot be ruled out if different primer sequences are used for different populations, the same primer sets had to be used. We finally obtained reliable MHC genotyping data from 97 individuals for DAB1 and 192 individuals for DAB3. Average number of reads per amplicon was 8569 ± 5495, ranging from 1040 to 22,155, in DAB1 and 9042 ± 8365, ranging from 311 to 78,094, in DAB3. The MHC sequences obtained in this study were deposited in NCBI GenBank under the accession numbers from MT943022 to MT943047.

### Tests of positive selection

The putative peptide-binding residues (PBRs) in the β1 domain region were identified based on the comparison with the sequences characterized in *Rhodeus sinensis*^21^. The strength of historical selection pressure acting on DAB was determined by the ratio of non-synonymous (*dN*) to synonymous (*dS*) substitutions (*ω*) based on Nei-Gojobori method^63^ with 2000 bootstrap replicates and modified under Juke–Cantor corrections. Tests of the positive selection signature for each of the codons were performed in two ways. First, codons were tested using CODEML implemented in PAMLX package^64,65^. The Bayes empirical Bayes (BEB) method was used to estimate Bayesian posterior probability (BPP). If the BPP was greater than 95%, the codon was considered to show significance. To obtain the phylogenetic tree included in the input (nekwick format) files for CODEML, Bayesian inference (BI) analysis was performed for the DAB1 or DAB3 sequences without consideration of outgroups using MrBayes 3.2.3 under the options of four heated chains, 40,000,000 generations and sampling tree at each 1000 generation^66^. *GTR* + *I* + *G* was chosen as the best-fit model for the BI analysis using jModelTest 2.0^67^ under Akaike Information Criterion (AIC)^68^. In CODEML, the likelihood ratio tests (LRT) were performed to compare the codon-based models, between M1a (nearly neutral) and M2a (positive selection), between M7 (beta distribution) and M8 (beta distribution and positive selection), and between M0 (one-ratio) and M3 (discrete). Second, the signature of positive selection was detected for each codon using the HyPhy package^69^ implemented in DataMonkey web server (http://www.datamonkey.org/), where three different codon-based maximum likelihood tests, FEL (Fixed Effects Likelihood), FUBAR (Fast Unconstrained Bayesian AppRoximation) and MEME (Mixed Effects Model of Evolution), were used. Phylogenetic relationship was reconstructed under the default setting in DataMonkey.

### Supertype classification

MHC allelic diversity at the intra-population level was quantified by estimating the allele number, haplotype diversity (*H*_d_) and nucleotide diversity (*π*) with DnaSP 5.10^70^. Pairwise *ϕ*_ST_ values were calculated among populations as an estimate of genetic differentiation using DnaSP^70^. The significance of the estimated *ϕ*_ST_ values was tested using 1000 permutations. Bayesian Structure analysis was performed for the *K* = 3 that was predicted to be the most reliable by Δ*K*^60^ implemented in Structure Harvester^61^, with ten independent MCMC runs, each consisting of 4 × 10^5^ generations after a burn-in of 10^5^. The input file for Structure analysis was constructed in a way that the corresponding alleles or supertypes for each individual were present (1) or not (0), or data was missing (− 1).

The DAB1 and DAB3 alleles were assigned into functional supertypes by analyzing amino acid polymorphism at the PBRs in the β1 domain region^21,71^. The PBRs of all DAB sequences were numerically characterized based on the physicochemical properties of each amino acid^26,72,73^, based on five metric descriptors: z1(hydrophobicity), z2 (steric bulk), z3 (polarity), z4 and z5 (electronic effects). A matrix was created for each of DAB1 and DAB3 with rows representing alleles and columns representing the physiochemical descriptors of z1–z5 for each PBR. The matrix for each DAB was used for the input in clustering of alleles by discriminant analysis of principle components (DAPC) implemented in the R package *adagenet* 1.4-0^74^. The optimal number of supertypes were estimated using R package *NbClust* 3.0 (https://cran.rstudio.com/bin/windows/contrib/3.5/NbClust_3.0.zip) under the majority rule.

## Supplementary Information

Supplementary Information.
