# Supplementary material for: Differential patterns of diversity at neutral and adaptive loci in endangered Rhodeus pseudosericeus populations
Source: Sci Rep. 2021 Aug 5;11:15953. doi: 10.1038/s41598-021-95385-w (PMC8342555; doi:10.1038/s41598-021-95385-w)
Supplement: Supplementary file 1 — Supplementary Information. [file 41598_2021_95385_MOESM1_ESM.docx]

**Supplementary Table S1.** Information of 24 microsatellite loci used in this study and the diversity estimates obtained from seven *Rhodeus pseudosericeus* populations on the Korean Peninsula. Data include locus, fluorescence labeling (F), repeat motif (Repeat), primer sequence (Sequence, from 5’ to 3’), annealing temperature (T, ^o^C), total number of alleles (*N*_A_), allelic richness (*A*_R_), observed (*H*_O_) and expected (*H*_E_) heterozygosities, fixation indices (*F*_IS_) and the result of statistical testing of Hardy-Weinberg equilibrium (HWE, probability). The HWE probability was highlighted with bold when the value was less than 0.002 (after Bonferroni correction).

| **Locus** | **F** | **Repeat** | **Sequence** | **T** | ***N*_A_** | ***A*_R_** | ***H*_O_** | ***H*_E_** | ***F*_IS_** | **HWE** |
| --- | --- | --- | --- | --- | --- | --- | --- | --- | --- | --- |
| *TS06* | FAM | (AG)22 | F: GAGAAAAGCTTCACTCGCTG | 58 | 11 | 7.666 | 0.498 | 0.573 | 0.198 | 0.002 |
|  |  |  | R: TATAGCTGGATGCCTCTACACC |  |  |  |  |  |  |  |
| *RU35* | NED | (AC)14 | F: CACCGCATGCTTCTTTAATATCCAG | 58 | 7 | 4.955 | 0.402 | 0.402 | -0.090 | 0.375 |
|  |  |  | R: ATGTAAAGTCCGCGTGCTTGTG |  |  |  |  |  |  |  |
| *TS07* | VIC | (AG)22 | F: GCACTGACGTGTCTACCTGTTA | 58 | 8 | 6.702 | 0.522 | 0.55 | 0.235 | 0.006 |
|  |  |  | R: GAAAGAACCACAGTGCCATC |  |  |  |  |  |  |  |
| *TS10* | PET | (CA)25 | F: GGTTTGGACTGCTTTCACAC | 58 | 5 | 4.672 | 0.501 | 0.518 | -0.044 | 0.138 |
|  |  |  | R: GTGGGCTAATCCACAACATC |  |  |  |  |  |  |  |
| *TS19* | FAM | (TG)28 | F: CTGGGGAAGTGTGCCTATTT | 58 | 22 | 12.505 | 0.540 | 0.632 | 0.086 | **0.000** |
|  |  |  | R: CATCACCACCACAAAACTCC |  |  |  |  |  |  |  |
| *RU573* | NED | (GT)20 | F: TTATGGTTCAGATGTGGCATTATT | 58 | 5 | 4.118 | 0.458 | 0.516 | 0.050 | 0.034 |
|  |  |  | R: TGTATGTGACCTCCAGTGCTG |  |  |  |  |  |  |  |
| *TS24* | VIC | (ATAG)9 | F: GTTTACCGAGTACCAGAGCAAC | 58 | 4 | 2.646 | 0.475 | 0.464 | -0.056 | 0.736 |
|  |  |  | R: GATCTGCAGTGCACGAGTAAC |  |  |  |  |  |  |  |
| *TS66* | PET | (AGAA)11 | F: CTGGGGTACAAACAGTGGAGT | 58 | 13 | 8.266 | 0.478 | 0.519 | 0.077 | 0.013 |
|  |  |  | R: CCTTCACGTCTGCCAGTAAT |  |  |  |  |  |  |  |
| *TS28* | FAM | (CTTT)11 | F: GCTGATCGTCATCTTTTCCC | 58 | 18 | 11.034 | 0.630 | 0.678 | 0.009 | 0.025 |
|  |  |  | R: ATTGCCAGGTGCAGACTTC |  |  |  |  |  |  |  |
| *TS18* | NED | (GT)22 | F: CAGGCTTAGTCTGCCTTTGA | 58 | 9 | 6.965 | 0.368 | 0.453 | 0.424 | **0.000** |
|  |  |  | R: GCTTTCGTACCGCTAATACG |  |  |  |  |  |  |  |
| *TS81* | VIC | (AGAA)11 | F: ACGAGTGCACATTCTGTGGT | 58 | 18 | 11.565 | 0.592 | 0.669 | 0.083 | 0.002 |
|  |  |  | R: GTTTCCTGCTCTGAAGGTGT |  |  |  |  |  |  |  |
| *TS64* | PET | (AGAA)11 | F: CATGCTGGAAAGCTCAAGTC | 58 | 18 | 9.339 | 0.577 | 0.647 | -0.057 | 0.004 |
|  |  |  | R: GTGAGGCAGAGTGTGGAACAT |  |  |  |  |  |  |  |
| *TS36* | FAM | (GTGA)10 | F: GACATCAACACATGCCAGC | 58 | 15 | 8.417 | 0.407 | 0.431 | -0.173 | 0.018 |
|  |  |  | R: AGACCCCGATTCTCATACTACC |  |  |  |  |  |  |  |
| *TS22* | NED | (AGAA)9 | F: CTGCCGTTTCACAGGCTA | 58 | 6 | 4.727 | 0.299 | 0.382 | 0.119 | **0.000** |
|  |  |  | R: TAAGAAGGCATCGGTCAGTC |  |  |  |  |  |  |  |
| *TS70* | VIC | (GATA)11 | F: CTGTTGCTGGCTGCTTTAGT | 58 | 18 | 10.614 | 0.682 | 0.682 | 0.070 | 0.203 |
|  |  |  | R: CACCCACAGGTCAAAAGACT |  |  |  |  |  |  |  |
| *TS79* | PET | (TTTC)10 | F: CTACTGACCACATACATGGACC | 58 | 8 | 5.803 | 0.478 | 0.524 | 0.448 | 0.010 |
|  |  |  | R: GCATTGATCCAGCTCACCT |  |  |  |  |  |  |  |
| *TS94* | FAM | (TTTC)11 | F: GATGGAACTCCGAATGGTC | 58 | 19 | 10.898 | 0.620 | 0.636 | -0.032 | 0.103 |
|  |  |  | R: GGTCTTCTGTGCAGGTGTATCT |  |  |  |  |  |  |  |
| *TS59* | NED | (TG)22 | F: TTTGGTGAGACTTGAGCTGC | 58 | 7 | 5.137 | 0.357 | 0.401 | 0.137 | 0.003 |
|  |  |  | R: GACCGAGCTGTATCATTCTCTC |  |  |  |  |  |  |  |
| *TS87* | VIC | (TATC)11 | F: GACAGCGACCACTATGTCAGT | 58 | 6 | 4.452 | 0.254 | 0.267 | -0.022 | 0.065 |
|  |  |  | R: GCTTTCTCAGACTTCCACCTGT |  |  |  |  |  |  |  |
| *TS99* | PET | (ATAG)10 | F: GCTGTGTGTGTGTAATGGACTC | 58 | 12 | 8.634 | 0.462 | 0.496 | 0.313 | 0.022 |
|  |  |  | F: TGACGCATCTGCTGTCTCTA |  |  |  |  |  |  |  |
| *TS27* | FAM | (CTGT)11 | F: AACCTCTCTCCACTCTTCACTG | 58 | 3 | 2.221 | 0.215 | 0.233 | -0.127 | 0.170 |
|  |  |  | R: GACTACATCTCTATGTGCGGTG |  |  |  |  |  |  |  |
| *TS91* | NED | (TATC)11 | F: CAGCAGGATTTTCAGGTCC | 58 | 14 | 8.417 | 0.526 | 0.550 | 0.088 | 0.268 |
|  |  |  | R: CTGTTGCAGGAGAGAACAGTG |  |  |  |  |  |  |  |
| *TS13* | VIC | (CT)22 | F: GAGTAGAACACATCCGCTGC | 58 | 5 | 3.103 | 0.332 | 0.312 | 0.081 | 0.832 |
|  |  |  | R: ATACGGAGGACCACCCTTATAG |  |  |  |  |  |  |  |
| *TS76* | PET | (ATAG)10 | F: GACTCCTGTGGGTGGTAAAGT | 58 | 24 | 14.630 | 0.841 | 0.751 | 0.127 | 0.917 |
|  |  |  | R: GAAATGCCAGTCATCTGCC |  |  |  |  |  |  |  |

**Supplementary Table S2.** The signature of positive selection (*d*_N_/*d*_S_) in β1 domain regions of DAB1 and DAB3. Data comprise the number of residues (Codon) and the results of *Z* test (*Z* and *P*).

|  |  | DAB1 |  |  |  | DAB3 |  |
| --- | --- | --- | --- | --- | --- | --- | --- |
|  | Codon | *Z* | *P* |  | Codon | *Z* | *P* |
| PBRs | 17 | 4.275 | < 0.001***** |  | 20 | 2.099 | 0.019* |
| Non-PBRs | 53 | -1.366 | 1.000 |  | 55 | 1.093 | 0.138 |
| Total | 70 | 0.265 | 0.631 |  | 75 | 2.656 | 0.004* |

**Supplementary Table S3.** Summary of models that were used to discover the signature of selection across codons of DAB1 β1 domain. Data for six different models comprise the logarithm of the likelihood (ln*L*), codons showing the signature of positive selection and number of parameters estimated in each model.

| Model | ln*L* | Codons showing the signature of positive selection | Parameter estimates | | |
| --- | --- | --- | --- | --- | --- |
| *M0* (One-ratio) | -996.54 | None | *ω* = 1.83 | | |
| *M1a* (Nearly neutral) | -951.20 | Not allowed | | *p*_0_ = 0.88  *p*_1_ = 0.12 | *ω*_0_ = 0.03  *ω*_1_ = 1.00 |
| *M2a* (Positive selection) | -898.49 | 9, 23, 27, 34, 35, 44, 50, 53,  57, 58, 64, 67, 71, 72, 74, 75 | | *p*_0_ = 0.95  *p*_1_ = 0.00  *p*_2_ = 0.05 | *ω*_0_ = 0.38  *ω*_1_ = 1.00  *ω*_2_ = 14.75 |
| *M3* (Discrete) | -896.39 | Not analyzed | | *p*_0_ = 0.93  *p*_1_ = 0.05  *p*_2_ = 0.01 | *ω*_0_ = 0.37  *ω*_1_ = 9.15  *ω*_2_ = 26.63 |
| *M7* (ß distribution) | -953.74 | Not allowed | | *p* = 0.01 | *q* = 0.09 |
| *M8* (ß & Positive selection) | -898.48 | 9, 23, 27, 34, 35, 44, 50, 53,  57, 58, 64, 67, 71, 72, 74, 75 | | *p*_0_ = 0.95  (*p*_1_ = 0.04)  *p* = 2.23 | *q* = 3.43  *ω* = 14.99 |

**Supplementary Table S4.** Summary of models that were used to discover the signature of selection across codons of DAB3 β1 domain. Data for six different models comprise the logarithm of the likelihood (ln*L*), codons showing the signature of positive selection and number of parameters estimated in each model.

| Model | ln*L* | Codons showing the signature of positive selection | Parameter estimates | | |
| --- | --- | --- | --- | --- | --- |
| *M0* (One-ratio) | -493.83 | None | *ω* = 2.69 | | |
| *M1a* (Nearly neutral) | -490.86 | Not allowed | | *p*_0_ = 0.66  *p*_1_ = 0.35 | *ω*_0_ = 0.00  *ω*_1_ = 1.00 |
| *M2a* (Positive selection) | -898.49 | 9, 27, 44, 53, 58, 64, 68, 83 | | *p*_0_ = 0.70  *p*_1_ = 0.26  *p*_2_ = 0.05 | *ω*_0_ = 1.00  *ω*_1_ = 1.00  *ω*_2_ = 34.59 |
| *M3* (Discrete) | -896.39 | Not analyzed | | *p*_0_ = 0.00  *p*_1_ = 0.97  *p*_2_ = 0.03 | *ω*_0_ = 0.00  *ω*_1_ = 2.18  *ω*_2_ = 70.63 |
| *M7* (ß distribution) | -953.74 | Not allowed | | *p* = 0.005 | *q* = 0.008 |
| *M8* (ß & Positive selection) | -898.48 | 9, 27, 44, 53, 58, 64, 68, 74, 83 | | *p*_0_ = 0.96  (*p*_1_ = 0.04)  *p* = 3.68 | *q* = 0.005  *ω* = 34.59 |

**Supplementary Table S5.** The likelihood ratio tests (LRT) performed to verify the signature of positive selection in each nested codon-based model comparison for DAB1 and DAB3 β1 domain.

|  |  | DAB1 |  |  |  | DAB3 |  |
| --- | --- | --- | --- | --- | --- | --- | --- |
|  | ln LRT | *df* | *P* |  | ln LRT | *df* | *P* |
| *M7* vs *M8* | 55.26 | 2 | < 0.001 |  | 12.16 | 2 | < 0.01 |
| *M1a* vs *M2a* | 52.71 | 2 | < 0.001 |  | 12.62 | 2 | < 0.01 |
| *M0* vs *M3* | 100.15 | 4 | < 0.001 |  | 15.87 | 4 | < 0.01 |

**Supplementary Table S6.** The frequency distribution of DAB1 and DAB3 alleles across *Rhodeus pseudosericeus* populations.

|  | Allele | Supertype | DC | MH | JJ | HC | GD | SG | JC |
| --- | --- | --- | --- | --- | --- | --- | --- | --- | --- |
| DAB1 | *Rhps-DAB1*01:01:01* | 6 | 0.20 | 0.58 |  |  |  |  |  |
|  | *Rhps-DAB1*02:02:03* | 2 | 0.26 | 0.21 |  |  |  |  |  |
|  | *Rhps-DAB1*02:08* | 2 |  | 0.05 |  |  |  |  |  |
|  | *Rhps-DAB1*03:03* | 6 | 0.42 | 0.08 |  |  |  |  |  |
|  | *Rhps-DAB1*03:13* | 6 |  | 0.08 |  |  |  |  |  |
|  | *Rhps-DAB1*04:01* | 4 |  |  |  |  | 1.00 |  |  |
|  | *Rhps-DAB1*04:02* | 4 |  |  |  |  |  | 0.10 |  |
|  | *Rhps-DAB1*05:01* | 4 |  |  |  |  |  | 0.05 |  |
|  | *Rhps-DAB1*06:01* | 7 |  |  |  |  |  | 0.05 |  |
|  | *Rhps-DAB1*06:02* | 7 |  |  |  |  |  | 0.03 |  |
|  | *Rhps-DAB1*07:01:01* | 3 |  |  |  |  |  | 0.17 |  |
|  | *Rhps-DAB1*07:01:02* | 3 |  |  | 0.04 |  |  |  | 0.09 |
|  | *Rhps-DAB1*07:02* | 3 |  |  | 0.04 |  |  |  |  |
|  | *Rhps-DAB1*07:03* | 3 |  |  |  |  |  |  | 0.05 |
|  | *Rhps-DAB1*08:01* | 1 |  |  |  | 0.25 |  | 0.03 |  |
|  | *Rhps-DAB1*09:01* | 5 |  |  |  | 0.50 |  |  |  |
|  | *Rhps-DAB1*09:02* | 5 |  |  |  |  |  |  | 0.06 |
|  | *Rhps-DAB1*09:03:01* | 5 | 0.12 |  | 0.92 | 0.25 |  | 0.50 | 0.43 |
|  | *Rhps-DAB1*09:03:02* | 5 |  |  |  |  |  |  | 0.31 |
|  | *Rhps-DAB1*09:04* | 5 |  |  |  |  |  | 0.07 |  |
|  | *Rhps-DAB1*09:05* | 5 |  |  |  |  |  |  | 0.06 |
| DAB3 |  |  |  |  |  |  |  |  |  |
|  | *Rhps-DAB3*01:01:01* | 1 |  |  | 0.68 | 0.78 | 0.22 |  |  |
|  | *Rhps-DAB3*02:28* | 1 | 0.91 | 1.00 |  |  |  |  |  |
|  | *Rhps-DAB3*03:08* | 2 |  |  | 0.32 | 0.22 | 0.73 | 0.79 | 1.00 |
|  | *Rhps-DAB3*04:01* | 2 |  |  |  |  | 0.05 | 0.21 |  |
|  | *Rhps-DAB3*05:01* | 1 | 0.09 |  |  |  |  |  |  |

**Supplementary Table S7.** Pairwise MHC genetic differentiation (*φ*_ST_) of seven *Rhodeus pseudosericeus* populations from the Korean Peninsula. Estimates of DAB1 appear above the diagonal and estimates of DAB3 appear below the diagonal. All comparisons were significantly different from zero (*P* < 0.05).

|  | DC | MH | Han River populations | | | | |
| --- | --- | --- | --- | --- | --- | --- | --- |
|  |  |  | JJ | HC | GD | SG | JC |
| DC |  | 0.121 | 0.723 | - | - | 0.504 | 0.691 |
| MH | 0.075 |  | 0.869 | - | - | 0.664 | 0.838 |
| JJ | 0.726 | 0.830 |  | - | - | 0.085 | 0.003 |
| HC | 0.754 | 0.857 | -0.002 |  | - | - | - |
| GD | 0.763 | 0.860 | 0.322 | 0.422 |  | - | - |
| SG | 0.879 | 0.967 | 0.627 | 0.706 | 0.172 |  | 0.067 |
| JC | 0.911 | 1.000 | 0.672 | 0.754 | 0.209 | 0.190 |  |

**Supplementary Table S8.** Frequency distribution of DAB1 and DAB3 supertypes obtained based on the physicochemical properties of PBRs across seven *Rhodeus pseudosericeus* populations.

| Loci | Supertype | DC | MH | JJ | HC | GD | SG | JC |
| --- | --- | --- | --- | --- | --- | --- | --- | --- |
| DAB1 | 1 |  |  |  | - | - | 0.033 |  |
|  | 2 | 0.260 | 0.263 |  | - | - |  | 0.130 |
|  | 3 |  |  | 0.083 | - | - | 0.167 | 0.093 |
|  | 4 |  |  |  | - | - | 0.150 |  |
|  | 5 | 0.120 |  | 0.917 | - | - | 0.567 | 0.778 |
|  | 6 | 0.620 | 0.737 |  | - | - |  |  |
|  | 7 |  |  |  | - | - | 0.083 |  |
| DAB3 | 1 | 1.000 | 1.000 | 0.679 | 0.776 | 0.226 |  |  |
|  | 2 |  |  | 0.321 | 0.224 | 0.774 | 1.000 | 1.000 |

**Supplementary Table S9.** Sequence list of primers used in the second PCR step of the MHC genotyping. GenoAmpi5XX and GenoAmpi7XX annal with the forward and reverse primers pf the first PCR step, respectively. A total of 96 PCR products constitute one set, and each product can be recognized by 96 different combinations made by selecting eight out of GenoAmpi5XX and twelve out of GenoAmpi7XX. In each primer, the 8 bp index sequence (underlined) was designed to allow the identification of the final PCR products per each individual after demultiplexing. The left part of the index sequence is the adaptor sequence allowing the final amplicons to hybridize to the MiSeq flowcells, and the right part is the region to bind the 5’end of the template generated by the first PCR.

| Primer name | Sequence (5’ → 3’) |
| --- | --- |
| GenoAmpi501 | AATGATACGGCGACCACCGAGATCTACACTATAGCCTACACTCTTTCCCTACACGAC |
| GenoAmpi502 | AATGATACGGCGACCACCGAGATCTACACATAGAGGCACACTCTTTCCCTACACGAC |
| GenoAmpi503 | AATGATACGGCGACCACCGAGATCTACACCCTATCCTACACTCTTTCCCTACACGAC |
| GenoAmpi504 | AATGATACGGCGACCACCGAGATCTACACGGCTCTGAACACTCTTTCCCTACACGAC |
| GenoAmpi505 | AATGATACGGCGACCACCGAGATCTACACAGGCGAAGACACTCTTTCCCTACACGAC |
| GenoAmpi506 | AATGATACGGCGACCACCGAGATCTACACTAATCTTAACACTCTTTCCCTACACGAC |
| GenoAmpi507 | AATGATACGGCGACCACCGAGATCTACACCAGGACGTACACTCTTTCCCTACACGAC |
| GenoAmpi508 | AATGATACGGCGACCACCGAGATCTACACGTACTGACACACTCTTTCCCTACACGAC |
| GenoAmpi509 | AATGATACGGCGACCACCGAGATCTACACTTGCTTGCACACTCTTTCCCTACACGAC |
| GenoAmpi510 | AATGATACGGCGACCACCGAGATCTACACGAGAGGTTACACTCTTTCCCTACACGAC |
| GenoAmpi511 | AATGATACGGCGACCACCGAGATCTACACACCTGGTTACACTCTTTCCCTACACGAC |
| GenoAmpi512 | AATGATACGGCGACCACCGAGATCTACACAAGCGGAAACACTCTTTCCCTACACGAC |
| GenoAmpi513 | AATGATACGGCGACCACCGAGATCTACACCGGAACAAACACTCTTTCCCTACACGAC |
| GenoAmpi514 | AATGATACGGCGACCACCGAGATCTACACGGTAAGCTACACTCTTTCCCTACACGAC |
| GenoAmpi515 | AATGATACGGCGACCACCGAGATCTACACTGTGGCATACACTCTTTCCCTACACGAC |
| GenoAmpi516 | AATGATACGGCGACCACCGAGATCTACACACTACGGAACACTCTTTCCCTACACGAC |
| GenoAmpi701 | CAAGCAGAAGACGGCATACGAGATCGAGTAATGTGACTGGAGTTCAGACGTG |
| GenoAmpi702 | CAAGCAGAAGACGGCATACGAGATTCTCCGGAGTGACTGGAGTTCAGACGTG |
| GenoAmpi703 | CAAGCAGAAGACGGCATACGAGATAATGAGCGGTGACTGGAGTTCAGACGTG |
| GenoAmpi704 | CAAGCAGAAGACGGCATACGAGATGGAATCTCGTGACTGGAGTTCAGACGTG |
| GenoAmpi705 | CAAGCAGAAGACGGCATACGAGATTTCTGAATGTGACTGGAGTTCAGACGTG |
| GenoAmpi706 | CAAGCAGAAGACGGCATACGAGATACGAATTCGTGACTGGAGTTCAGACGTG |
| GenoAmpi707 | CAAGCAGAAGACGGCATACGAGATAGCTTCAGGTGACTGGAGTTCAGACGTG |
| GenoAmpi708 | CAAGCAGAAGACGGCATACGAGATGCGCATTAGTGACTGGAGTTCAGACGTG |
| GenoAmpi709 | CAAGCAGAAGACGGCATACGAGATCATAGCCGGTGACTGGAGTTCAGACGTG |
| GenoAmpi710 | CAAGCAGAAGACGGCATACGAGATTTCGCGGAGTGACTGGAGTTCAGACGTG |
| GenoAmpi711 | CAAGCAGAAGACGGCATACGAGATGCGCGAGAGTGACTGGAGTTCAGACGTG |
| GenoAmpi712 | CAAGCAGAAGACGGCATACGAGATCTATCGCTGTGACTGGAGTTCAGACGTG |
| GenoAmpi713 | CAAGCAGAAGACGGCATACGAGATAGGAGGAAGTGACTGGAGTTCAGACGTG |
| GenoAmpi714 | CAAGCAGAAGACGGCATACGAGATAGCAAGCAGTGACTGGAGTTCAGACGTG |
| GenoAmpi715 | CAAGCAGAAGACGGCATACGAGATTCATCACCGTGACTGGAGTTCAGACGTG |
| GenoAmpi716 | CAAGCAGAAGACGGCATACGAGATCGTAGGTTGTGACTGGAGTTCAGACGTG |
| GenoAmpi717 | CAAGCAGAAGACGGCATACGAGATTCAGATCCGTGACTGGAGTTCAGACGTG |
| GenoAmpi718 | CAAGCAGAAGACGGCATACGAGATCGTGATCAGTGACTGGAGTTCAGACGTG |
| GenoAmpi719 | CAAGCAGAAGACGGCATACGAGATAGTCGCTTGTGACTGGAGTTCAGACGTG |
| GenoAmpi720 | CAAGCAGAAGACGGCATACGAGATGAACGCTTGTGACTGGAGTTCAGACGTG |
| GenoAmpi721 | CAAGCAGAAGACGGCATACGAGATTACGCCTTGTGACTGGAGTTCAGACGTG |
| GenoAmpi722 | CAAGCAGAAGACGGCATACGAGATCTCATCAGGTGACTGGAGTTCAGACGTG |
| GenoAmpi723 | CAAGCAGAAGACGGCATACGAGATTCTTCTGCGTGACTGGAGTTCAGACGTG |
| GenoAmpi724 | CAAGCAGAAGACGGCATACGAGATGCTGGATTGTGACTGGAGTTCAGACGTG |

**Supplementary Figure S1.** The reliable numbers of genetically distinguishable clusters (*K*) predicted by Δ*K* implemented in Structure Harvester for microsatellite, MHC allelic and MHC supertype data.
